# Effect of ultrasonic vibration on forming force and forming quality in micro-punching with a flexible punch

**DOI:** 10.1038/s41598-022-21236-x

**Published:** 2022-10-07

**Authors:** Chang-tao Liu, Xiao-guang Xu, Dong Zhang, Feng Luo, Li-kuan Zhu

**Affiliations:** 1 Shenzhen Key Laboratory of High Performance Nontraditional Manufacturing, College of Mechatronics and Control Engineering, Shenzhen University, Shenzhen, 518060 China; 2 Shenzhen Customs Industrial Products Testing Technology Center, Shenzhen, 518045 China

**Keywords:** Engineering, Mechanical engineering

## Abstract

In this paper, a special ultrasonic microforming method, Micro Ultrasonic thin Sheetmetal Forming using molten plastic as a flexible punch (short as Micro-USF), was used to conduct micro-punching experiments on stainless steel sheet with thickness of 10 μm and 20 μm. The influence of ultrasonic vibration on forming force and forming quality were investigated. The experimental results showed that the forming force required for punching thin sheet metal decreased gradually as the ultrasonic time or ultrasonic power increased. By applying the ultrasonic vibration effect, the forming force could be decreased dramatically and the maximum value of forming force drop could reach 86%. Moreover, with the application of ultrasonic vibration, the size accuracy and shape accuracy of micro-holes could be increased by 36.92% and 22.65%, but the cross-section quality of micro-holes were not significantly improved.

## Introduction

Micro-punching has been widely used in industrial production, because of its the advantages of simple process, high processing efficiency and low cost. In traditional mechanical micro-punching, there are some problems such as the easy wear of micro-punch and the difficult alignment between punch and die^1^. However, it was found that the forming quality of workpiece could be improved by applying the ultrasonic vibration^2^. With the development of micro-forming technology in recent years, the influence of ultrasonic on the micro-punching has been paid more and more attention.

Wang et al. punched T2 copper sheets of 100 μm and 200 μm respectively and applied high-frequency vibration of 1.0 kHz on the punch, and obtained square holes with sizes of 500 μm × 500 μm and 2 mm × 2 mm respectively^3,4^. Then the effect of forming force and the vibration on crack formation and cross-section quality was investigated. The results showed that the forming force was decreased by 5% and the proportion of bright zone in the cross-section increased with the help of high frequency vibration. Witthauer et al. added transverse vibration on the punch and carried out a punching experiment on the 3.2 mm circular plates using a thickness of 800 μm aluminum sheet^5^. With the effect of the vibration, the forming force decreased by 30% and the area of the fracture zone decreased from 43% to 24%. Yeh et al. studied the effect of ultrasonic vibration on shearing load and the micro-hole edge contour and found that the average punching load was reduced by 12% and the burr height was also decreased^6^. Mita et al. applied ultrasonic vibration with a frequency of 60.5 kHz on the punch, and conducted the punching experiment on the stainless steel sheet with 100 μm m thickness^7^. The results showed that the shear load decreased with the ultrasonic amplitude increasing and the maximum shear load could be reduced by 60%, which indicated that vibration could reduce the forming force and improve the cross-section quality of punching workpieces. J. Sun et al. applied ultrasonic vibration on the rigid punch and the end of the rigid punch was opened with a micro-concave die needed for punching^8^. The polyurethane pad was used as the flexible punch and a round hole with diameter of 800 μm and a square hole with size of 800 μm × 800 μm were punched on a brass sheet with the thickness of 50 μm. The results showed that ultrasonic vibration effect could increase the probability of punching success. Considering there is limited knowledge on the influence of vibration on forming force in flexible punch punching, this paper applied Micro Ultrasonic thin Sheetmetal Forming using molten plastic as a flexible punch (Micro-USF)^9^ and mainly discuss the influence of ultrasonic vibration on forming force and forming quality in the ultrasonic micro-punching with a flexible punch.

## Micro-USF forming principle and research method

### Micro-USF forming principle

**Figure 1:**
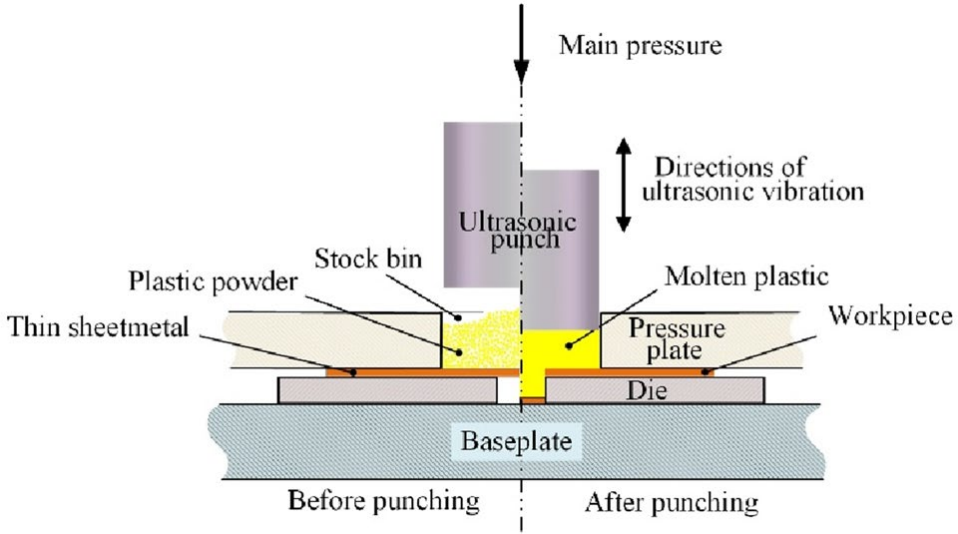
Schematic diagram of Micro-USF forming principle.

As shown in Fig. 1, the stock bin was filled with plastic powder before the experiment. In the experiment, the descending ultrasonic head exerted down ward pressure and ultrasonic vibration with a frequency of 20 kHz on the plastic powder. At the effect of ultrasonic punch pressure and ultrasonic vibration, the collision and friction behaviors of plastic powder particles could generate heat to melt the plastic powder into a viscous fluid medium (molten plastic), which acted as a flexible punch to transfer the ultrasonic head pressure and ultrasonic vibration to the thin sheetmetal. At the effect of ultrasonic head main pressure and ultrasonic vibration, the thin sheetmetal was cut off by the edge around the micro-hole of the die, and the micro-punching sample could be obtained. In Micro-USF, the ultrasonic vibration could melt the plastic powder to obtain a flexible punch. On the other hand, ultrasonic vibration was also transmitted to the thin sheetmetal through the molten plastic to assist the punching process.

### Research method

When investigating the effect of ultrasonic vibration on the punching forming force and its forming quality, for rigid punch, the analysis can be performed directly by whether ultrasonic was applied or not^3,5–7^. However, in the flexible punch forming like Micro-USF, the ultrasonic vibration converts the plastic powder into a viscous flow state of the flexible punch. Therefore, the effects produced by ultrasonic cannot be studied simply by comparing the two cases of applying ultrasonic and not applying ultrasonic. It is necessary to arrange an experiment without applying ultrasonic under a viscous flow state flexible punch as a comparison with the Micro-USF method. In this paper, we designed a device to melt plastic powder by heating in order to perform micro-punching experiments without ultrasonic.

## Experimental equipment and materials

### Experiment platform

**Figure 2:**
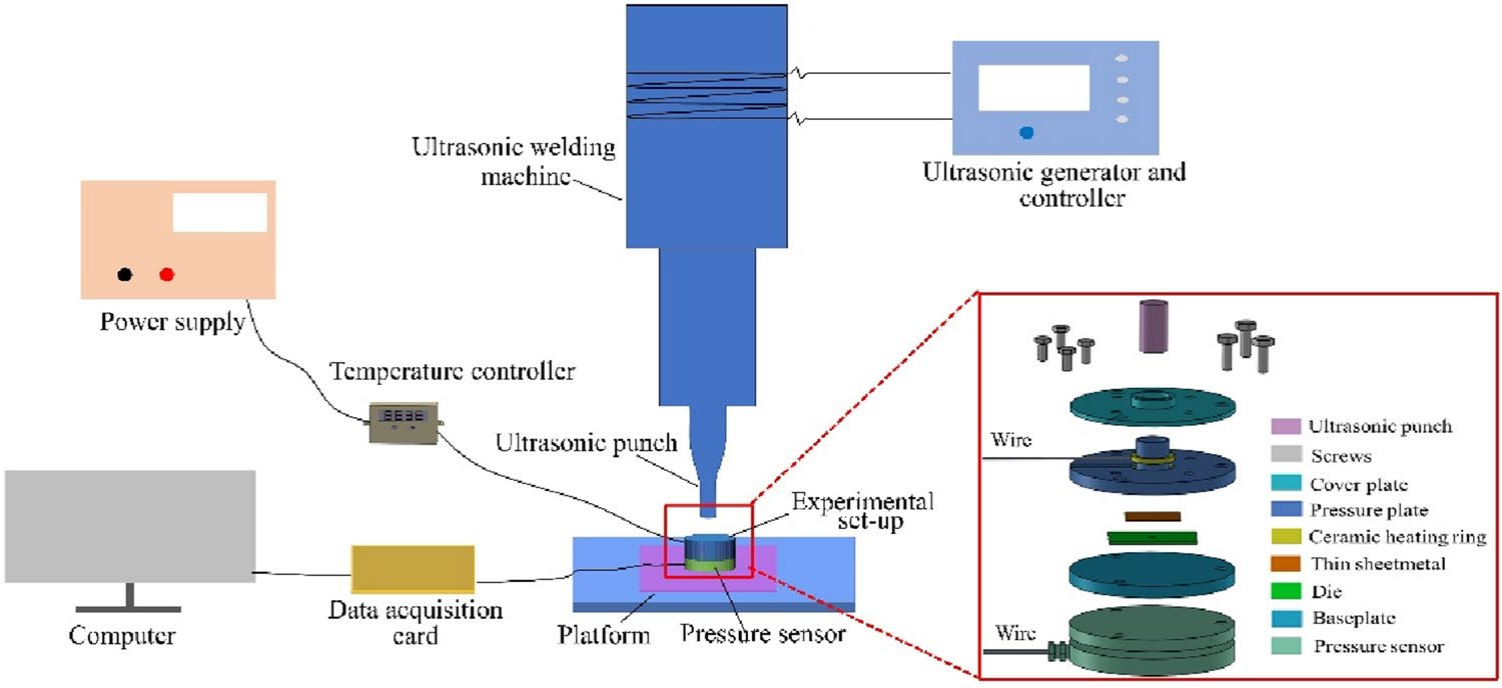
Schematic diagram of Micro-USF micro-punching experimental platform.

**Figure 3:**
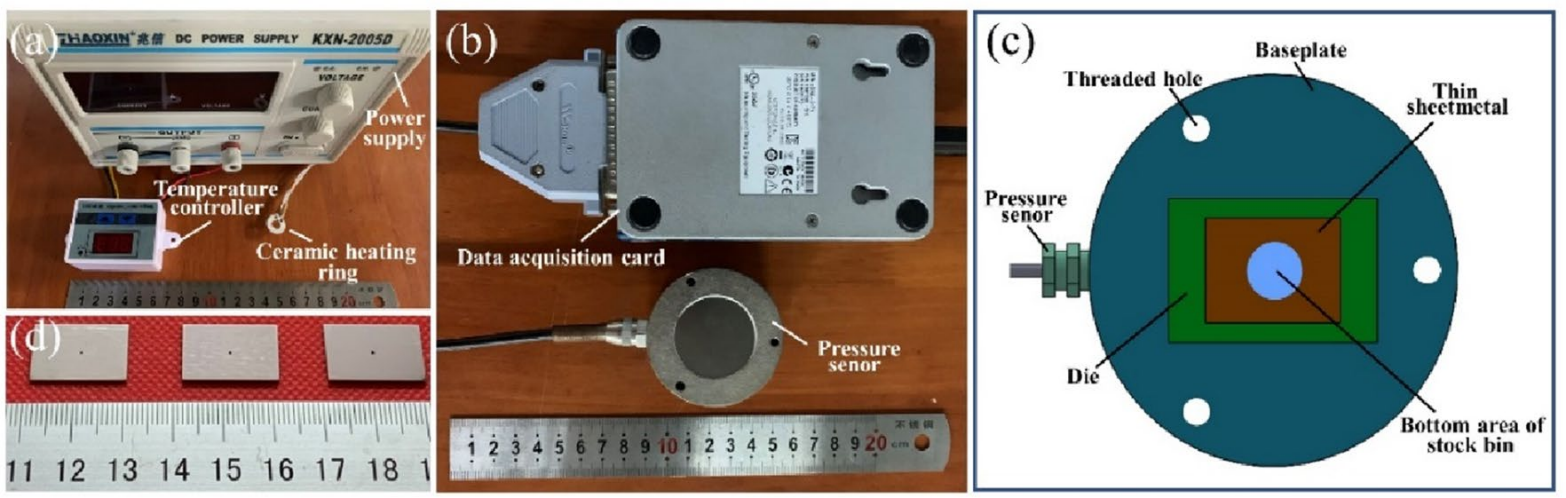
Physical diagram of the experimental equipment and the pressure sensor force measurement diagram. **(a)** physical view of heating module parts. **(b)** pressure acquisition module physical drawing. **(c)** schematic diagram of pressure sensor force measurement**. (d)** die physical picture.

As shown in Fig. 2, the experiment platform for the Micro-USF consisted of ultrasonic plastic welder, Experimental set-up, temperature controller and data collector, etc. As shown in Fig. 3a, the temperature controller (XH-W3001, Zhibao Electronics Co., LTD, China), power supply (KXN-2005D, Shenzhen zhaoxin Electronic Equipment Co., LTD, China) and ceramic heating ring ( YPS-JRB3825, Upps Technology Co., LTD, China) constituted the heating module for the non-ultrasonic punching experiment. The data collector and pressure sensor constituted a pressure acquisition module. The main parameters for the ultrasonic plastic welder were shown Table 1 and its internal cylinder would provide the main pressure of ultrasonic punch and ultrasonic vibration generated by ultrasonic generation and controller.

**Table 1:** Main parameters of ultrasonic plastic welder.

parameter	Ultrasonic frequency	Maximum power	Ultrasonic power	Cylinder air pressure	Ultrasonic vibration time
Numerical range	20 kHz	2000 W	50% ~ 100%	0.08 ~ 0.7 MPa	0 ~ 9.99 s

### Microforming device

In order to carry out the Micro-USF punching experiment and non-ultrasonic punching experiment, a dual-use experimental device was designed in this paper (Fig. 2). The experimental device was mainly composed of pressure plate, ceramic heating ring, baseplate and pressure sensor. The round hole in the center of the pressure plate was used as a stock bin for storing plastic powder. The diameter of the stock bin space is 5.6 mm and the depth is 5.1 mm. Ceramic heating rings surround the pressure plate and could melt the plastic powder in the stock bin. A temperature sensor was placed near the side wall of the stock bin to monitor the temperature in real time. When the non-ultrasonic experiment was carried out, the plastic powder was melted by heating and the thin sheet metal was punched. In the Micro-USF device, the heating device did not work and the plastic powder was melt by applying ultrasonic vibration and then the thin sheetmetal was punched.

### Pressure acquisition module

The pressure sensor was placed under the baseplate, and the thin sheetmatal, die, baseplate and pressure sensor were rigidly connected. The pressure acquisition module mainly included pressure sensor and data acquisition card. Pressure sensor (QLMH-P, Bengbu Qili sensor, China) and multi-channel data acquisition card (NI9237, National Instruments, America) were shown in the Fig. 3b. As shown in Fig. 3c, the pressure sensor measured the force exerted on the thin sheetmetal surface in relation to the bottom area of stock bin. The sampling frequency of the forming force was 2 kHz.

### Die and experimental observation equipment

On the Cr12MoV die steel plate with thickness of 1 mm, the circular through holes with diameters of 500,600,700 μm were manufactured fisrtly, which were used as micro-punching die. The actual die for the experiment is shown in Fig. 3d.

Electron microscope (Quanta 450 FEG, FEI, America) was used to characterize the surface and cross-section of micro-punching samples.

### Experimental materials

X10CrNi18-8 cold-rolled stainless steel with thickness of 10 μm and 20 μm was used as the experimental material. Its chemical composition is shown in Table 2.

**Table 2:** X10CrNi18-8 stainless steel chemical composition.

Chemical composition	C	Si	Mn	P	S	N	Cr	Mo	Ni
Mass fraction (%)	0.05–0.15	≤ 2.00	≤ 2.00	≤ 0.045	≤ 0.015	≤ 0.11	16.00–19.00	0.80	6.00 – 9.50

Tensile machine (Roell Z050, Zwick, Germany) was used to stretch X10CrNi18-8 stainless steel sheet, and the mechanical properties of the experimental material were measured as shown in Table 3.

**Table 3:** Mechanical properties of 10 μm and 20 μm thick X10CrNi18-8 stainless steel sheet.

Sheet thickness ( μm)	Modulus of elasticity (E/GPa)	Yield strength (MPa)	Tensile strength (MPa)
10	172.82	1356.35	1462.67
20	180.77	1379.19	1425.00

EVA (Ethylene Vinyl Acetate copolymer) plastic powder was used as the raw material of flexible punch. The average particle size of EVA plastic powder is 350 μm, and the melting temperature is 90 °C.

## Experiment

The thin sheet metal was micro-punched using the microforming device (Fig. 2) and the die (Fig. 3). The Micro-USF punching experiment and the micro-punching with heating molten plastic powder without ultrasonic (non-ultrasonic punching experiment) were included in this manuscript. Both experiments were completed on the same experimental platform described above. If the ultrasonic time of the ultrasonic welder was 0 s, it meant that no ultrasonic vibration was used in the punching experiment. In all experiments, the delay time and pressure holding time of ultrasonic welder were set as 4.0 s and 2.0 s, respectively^10^.

### Determination of punching forming force

In the experiment, a high cylinder pressure was selected to ensure that round holes could be punched on the thin sheetmetal. Then successively decrease the cylinder pressure, until three consecutive punching could not fabricate a round hole on the thin sheetmetal. At this point, the force generated by the air pressure in the previous cylinder was considered as the minimum forming force that could finish punching on the thin sheetmetal. For each group of parameters in this paper, the minimum punching forming force was obtained according to the above method. The forming force appearing later was the minimum forming force that could be punched exactly on a thin sheetmetal.

### Non-ultrasonic punching experiment

In the non-ultrasonic punching experiment, the heating temperature of plastic powder was set 95 °C which was 5 °C above EVA melting temperature. According to the method described “Determination of punching forming force” in Section, the value of forming force could be determined stainless steel sheets with thickness of 10 μm and 20 μm, which was used for comparison with the forming force in the Micro-USF punching experiment.

### Micro-USF punching experiment

According to the method described  “Determination of punching forming force” in Section the forming force value of thin sheetmetal could be obtained by adjusting ultrasonic time and ultrasonic power in Micro-USF punching experiment. It was found that under the action of ultrasonic vibration, the thin sheetmetal would crack due to the larger values of ultrasonic forming parameters and the forming force values were not recorded at those condition. As shown in Table 4, the valid range for the ultrasonic forming parameters was obtained to obtain the forming force of thin sheetmetal punching in the Micro-USF experiment. In the experiment, the interval of ultrasonic time change was 0.1 s and the interval of ultrasonic power change was 5%.

**Table 4:** Ultrasonic forming parameters of thin sheetmetal without fracture in Micro-USF experiment.

Thinsheetmetal(μm)	Die diameter (μm)	Ultrasonic power (%)	Ultrasonic time (s)
10	500–700	50	0–0.3
		55	0–0.2
		60	0–0.1
20	700	50	0–0.4
		55	0–0.2
		60	0–0.1
		65	0–0.1

## Results and discussion

### Effect of ultrasonic vibration on forming force

**Figure 4:**
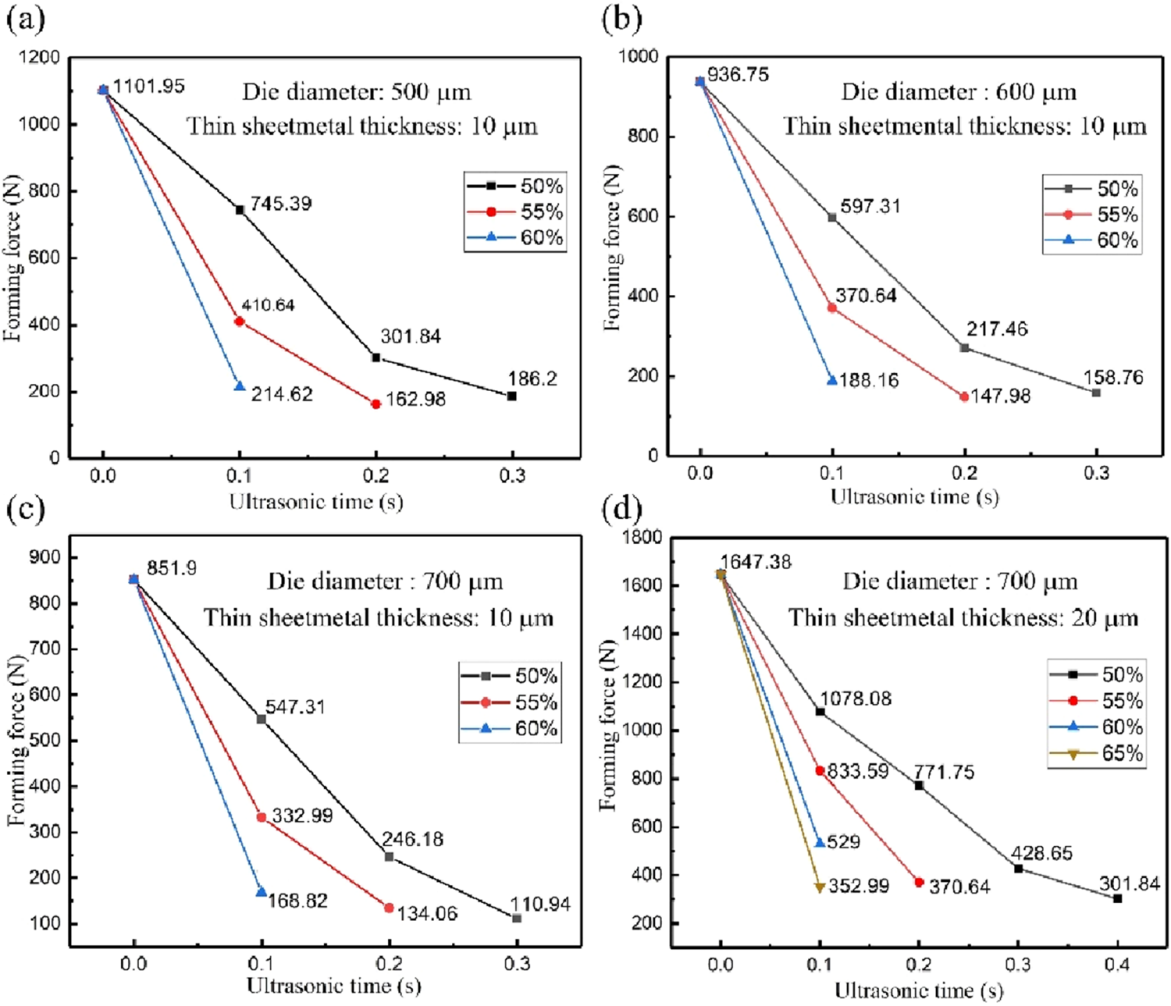
Forming forces obtained for different thicknesses of thin sheet metal and different diameters of dies with different ultrasonic forming parameters. **(a)** Thin sheetmetal:10 μm, die diameter: 500 μm. **(b)** Thin sheetmetal:10 μm, die diameter: 600 μm. **(c)** Thin sheetmetal:10 μm, die diameter: 700 μm. **(d)** Thin sheetmetal: 20 μm, die diameter: 700 μm.

The forming force obtained by Micro-USF punching experiment and non-ultrasonic punching experiment for stainless steel sheet with thickness of 10 μm and 20 μm on the die with diameter of 500, 600 and 700 μm was shown in Fig. 4.

In Fig. 4, 0 s of ultrasonic time indicated the forming force obtained in the non-ultrasonic punching experiment. It could be seen from Fig. 4 that ultrasonic had obvious effect on the forming force in the thin sheetmetal punching. By applying ultrasonic vibration, the forming force in the thin sheetmetal punching decreased greatly with the increasing of ultrasonic time and ultrasonic power. For example, the forming force of the thin sheetmetal was 1101.95 N in the non-ultrasonic punching experiment for a thin sheetmetal with 10 μm thickness and a die with 500 μm thickness. However, in the Micro-USF punching experiment, this forming force was decreased by 32.36% when ultrasonic power was 50% and the ultrasonic time was 0.1 s. When the ultrasonic time and ultrasonic power were 0.20 s and 55% respectively, the forming force of thin sheetmetal punching reached the minimum value of 162.98 N. At this time, compared with the forming force obtained without ultrasonic punching experiment, the forming force in the thin sheetmetal punching could be dropped by 85.21%. As shown in Table 5, the maximum decreasing amplitudes of forming forces in the thin sheetmetal punching with 10 μm and 20 μm thickness and die diameters of 500 μm, 600 μm and 700 μm were determined. The results showed that the punching forming force of 10 μm and 20 μm thin sheetmetals on the 500–700 μm die decreased by 85.21%, 84.2%, 86.98% and 81.68% in the Micro-USF punching experiment, respectively. For thin sheetmetal punching with thickness of 10 μm, the maximum decreasing amplitude of forming force is similar due to the effect of ultrasonic vibration and the deviation was less than 3%. However, for thin sheetmetals with different thickness, the maximum reducing amplitude of punching forming force of thin sheetmetals of 10 μm thickness was 5.3% larger than that of thin sheetmetals of 20 μm thickness under the action of ultrasonic. This was mainly because that the punching forming force in the non-ultrasonic punching experiment was about twice in 20 μm thick sheet than that in the 10 μm thick sheet. However, in the Micro-USF punching experiment, with the increasing of ultrasonic forming parameters, the thin sheetmetal forming force would decrease and the thin sheetmetal would become more prone to rupture. For the thin sheetmetal of 20 μm thickness in Micro-USF punching, the thin sheetmetal had broken when the forming force of thin sheetmetal punching dropped to about 86.98%, which indicated that the forming force of thin sheetmetal punching was invalid.

**Table 5:** Maximum drop of ultrasonic on forming force of thin sheetmetal punching.

Thin sheetmetal( μm)	Die diameter ( μm)	Ultrasonic time (s)	Ultrasonic power (%)	Decrease amplitude of forming force (%)
10	500	0.20	55	85.21
10	600	0.20	55	84.20
10	700	0.30	50	86.98
20	700	0.40	50	81.68

### Effect of ultrasonic vibration on forming quality

The thin sheetmetal of 20 μm was punched on the die with 700 μm diameter at the condition of no ultrasonic and ultrasonic vibration time of 0.4 s with an ultrasonic power of 50%, respectively. Six samples were prepared in the non-ultrasonic punching experiment and the Micro-USF punching experiment, respectively. Three samples were randomly selected to analyze the effect of ultrasonic on the micro-hole forming accuracy and the other three samples were used to investigate the effect of ultrasonic on the micro-hole cross-section quality.

#### Forming accuracy

**Figure 5:**
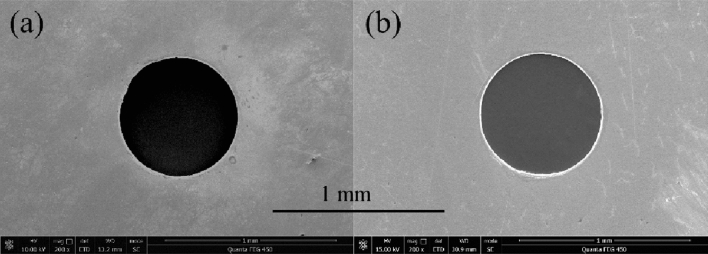
Scanning electron micrograph of the surface of the sample obtained from Micro-punching experiment. **(a)** Non-ultrasonic punching experiment. **(b)** Micro-USF punching experiment.

The evaluation method proposed by Liu et al.^11^ was adopted to evaluate the size accuracy and shape accuracy of the three samples obtained under the non-ultrasonic punching experiment and Micro-USF punching experiment, respectively (Fig. 5). The results were shown in Table 6 below.

**Table 6:** Diameter error and roundness error of samples obtained with and without ultrasonic vibration.

Type of experiment	Size errors( μm)	Roundness errors( μm)
Non-ultrasonic punching experiment	11.51	4.90
Micro-USF punching experiment	7.26	3.79

As can be seen from Table 6 above, sample size errors and roundness errors were 7.26 μm and 3.79 μm at Micro-USF punching experiment. The size errors and roundness errors of samples were 11.51 μm and 4.90 μm at the non-ultrasonic punching experiment. From the above experimental results, compared with the result at the non-ultrasonic punching experiment, the sample size accuracy and shape accuracy obtained by Micro-USF punching experiment are improved by 36.92% and 22.65%, respectively. It showed that the size accuracy and shape accuracy of the sample could be improved with the effect of the ultrasonic vibration.

#### Cross-section quality

**Figure 6:**
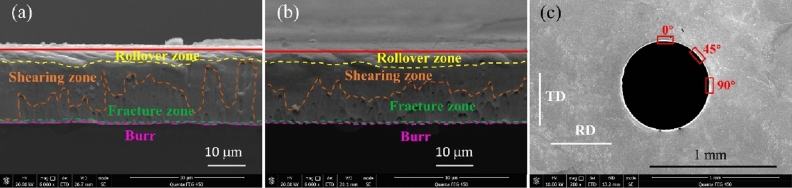
Micro-hole cross-section and its observation position. **(a)** Micro-hole cross-sections in non- ultrasonic punching experiments. **(b)** cross-sectional view of micro-hole in Micro-USF punching experiment. **(c)** micro-hole cross-section observation position.

Scanning electron microscope (SEM) was used to observe the cross-section morphology of the samples in non-ultrasonic punching experiment and Micro-USF punching experiment respectively, and the results were as shown in Fig. 6a,b. The results showed that micro-hole cross-sections could be generally divided into rollover zone, shearing zone, fracture zone and burr for both the non-ultrasonic punching and Micro-USF punching experiment. The rolled stainless steel sheet had anisotropy in this paper. In order to evaluate the difference of micro-holes cross-section morphology between non-ultrasonic punching experiment and Micro-USF punching experiment, cross-sections near three typical positions with rolling directions of 0°, 45° and 90° were observed in this paper. The observed positions were shown in the red box in Fig. 6c. In Fig. 6c, RD(Rolling Direction) was the rolling direction of stainless steel sheet, TD was the direction perpendicular to RD, and the angle value represented the included angle between the cross-section and RD direction. Scanning electron microscope (SEM) was used to observe the cross-section morphology of three samples obtained by non-ultrasonic punching experiment and Micro-USF punching experiment, respectively. RZ (Rollover Zone) was used to represent the rollover zone of micro-holes cross-section. SZ (Shearing Zone) indicated the shearing zone of the micro-holes cross-section. FZ (Fracture Zone) indicated the fracture zone of the micro-holes cross-section. Bur. indicated the burr of the micro-holes cross-section. The proportion of each zone of the cross-section was shown in Table 7.

**Table 7:**
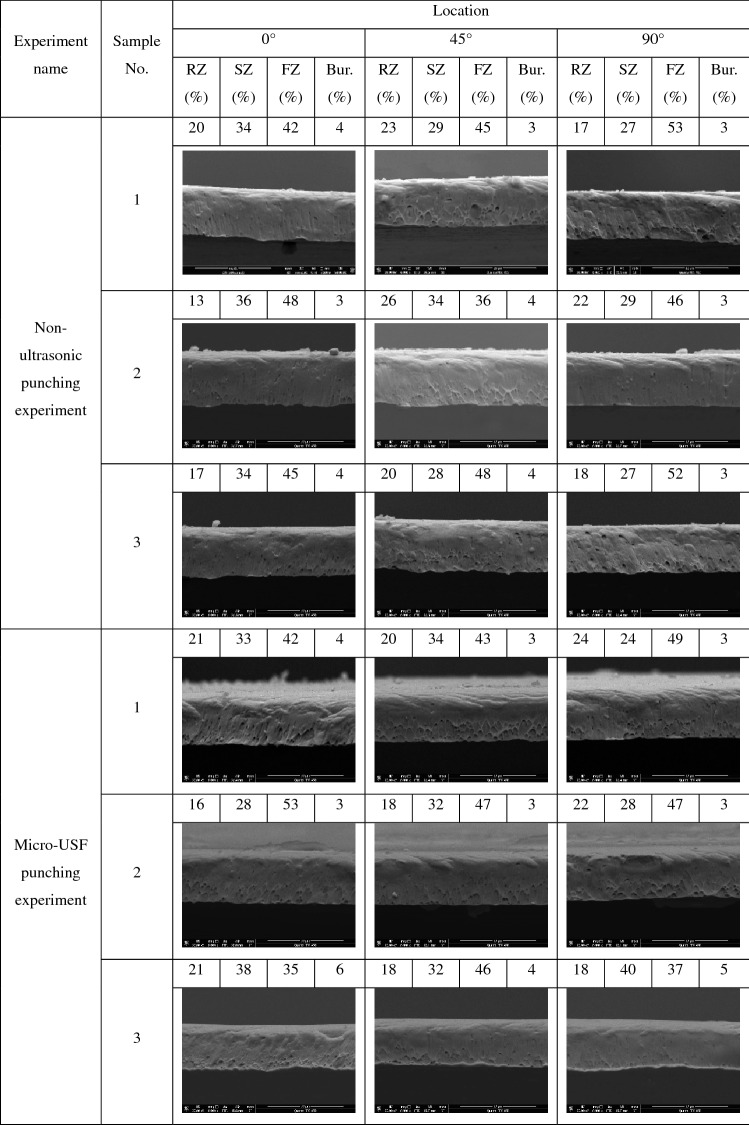
The proportion of each zone at 0°, 45° and 90° of micro-holes cross-section.

Since there was no significant dividing line between cross-sections, the percentage of width of each cross-section given was only a rough value in Table 7. By comparing the photos in Table 7, it could be seen that no matter in the non-ultrasonic punching experiment or the Micro-USF punching experiment, even at the same angle position, the cross-sections of the three samples had great differences in each zone. The cross-section morphology could be related to the local shear force and shear velocity. In punching with molten EVA as a flexible punch, the fracture around the round hole was not simultaneous, but in a certain sequence. The magnitude and direction of the shear force on the first and second parts of the fracture were different^9^ and the fracture velocity was also different. Because the location of fracture before and after was random in different samples, different samples would show differences even if the cross-section was observed at the same angle. Generally, the sample in Micro-USF punching experiment had no significant quality improvement.

## Conclusions

In this paper, the influence of ultrasonic vibration on forming force and forming quality of parts in Micro-USF punching experiment was analyzed. The main conclusions were as follows:

It is proposed to obtain molten EVA plastic by heating method and use it as a flexible punch to realize ultrasonic contrast punching experiment. Through this contrast experiment, the influence of ultrasonic in Micro-USF punching could be studied.

Compared with Micro-USF punching experiment with ultrasonic and without ultrasonic, it was found that ultrasonic could greatly reduce the forming force and improve the size and shape accuracy of punching parts, but applying ultrasonic could not change the quality of punching surface significantly.

At the experimental conditions of ultrasonic time of 0.30 s and ultrasonic power of 50%, the forming force could be decreased by 86.98% when the thin sheetmetal with 10 μm thickness was punched on the die with 700 μm diameter. With ultrasonic time of 0.40 s and ultrasonic power of 50%, the size accuracy and shape accuracy of micro-holes were increased by 36.92% and 22.65% respectively when the thin sheetmetal with 20 μm was punched on the die with 700 μm diameter.

## Data Availability

All data generated or analyzed in this study are included in the present article.

## Abbreviations

Micro-USF: Micro Ultrasonic thin Sheetmetal Forming using molten plastic as a flexible punch
RD: Rolling direction
TD: Transverse direction
RZ: Rollover Zone
SZ: Shearing Zone
FZ: Fracture Zone
Bur: Burr
